# RNA Binding to CCRRM of PABPN1 Induces Conformation Change

**DOI:** 10.3390/biology14040432

**Published:** 2025-04-17

**Authors:** Shengping Zhang, Ting Chen, Yunlong Zhang, Changrui Lu

**Affiliations:** College of Biological Science and Medical Engineering, Donghua University, Shanghai 201620, China; zhangshengping2018@163.com (S.Z.); chenting@dhu.edu.cn (T.C.); zhyl@dhu.edu.cn (Y.Z.)

**Keywords:** RBPs, PABPN1, RNA binding, SAXS, SHAPE

## Abstract

Poly(A) Binding Protein Nuclear 1 (PABPN1) is a nuclear poly(A)-binding protein that is highly conserved in eukaryotes. It plays multifaceted roles in RNA processing and metabolism, with its dysregulation closely linked to various diseases. PABPN1 contains an alanine-rich N-terminus, a central coiled-coil domain (CC), a conserved RNA recognition motif (RRM) and a C-terminal extension. PABPN1 influences mRNA splicing and stability through its RNA-binding capabilities, thereby modulating gene expression. While PABPN1 is known to interact with RNA, the molecular mechanism underlying this interaction with RNA awaits further investigation. Here, we designed and purified a PABPN1 fragment encompassing the RNA-binding domain (CCRRM fragment, amino acids 114–254). Using a combination of 3D modeling, small-angle X-ray scattering (SAXS) and selective 2′-hydroxyl acylation analyzed by primer extension (SHAPE) assay, our result indicated that CCRRM exhibits a high affinity for poly(A) RNA, a moderate affinity for GU-rich and CU-rich sequences, and negligible binding to AU-rich and CA-rich sequences. RNA binding induces conformation change in the CC. These results suggest that PABPN1 could potentially be involved in cytoplasmic polyadenylation and may influence the regulation of mRNA translation and degradation, although further investigation is required to confirm this role.

## Simple Summary

PABPN1 is a highly conserved nuclear poly(A)-binding protein in eukaryotes. This study utilized electrophoretic mobility shift assays, biolayer interferometry, and selective 2′-hydroxyl acylation analyzed by primer extension to confirm the RNA-binding capacity. We found that the CCRRM fragment of PABPN1 exhibits high affinity for poly(A) RNA, displays moderate affinity for GU-rich and CU-rich sequences, and shows minimal binding to AU-rich and CA-rich sequences. Furthermore, small-angle X-ray scattering was employed to analyze the solution conformations of CCRRM in its RNA-free and RNA-bound states. The results revealed that RNA binding induces conformational changes, primarily in the CC region. This local structural adjustment may enhance the protein’s ability to interact with RNA, thereby improving binding specificity.

## 1. Introduction

mRNA processing, also known as post-transcriptional regulation, is essential throughout the mRNA life cycle. This process encompasses various stages, including capping, cleavage, 3′-end processing, nuclear export, localization, and translation, all of which are meticulously regulated by RNA binding proteins (RBPs) [1]. These proteins specifically recognize and bind to RNA, serving a pivotal function in RNA metabolism and the regulation gene expression [2]. Mutations or dysfunctions in RBPs may lead to diseases. For instance, mutations in the PABPN1 gene have been linked to oculopharyngeal muscular dystrophy (OPMD), an autosomal dominant neuromuscular disease caused by an abnormal N-terminal alanine extension [3,4,5,6,7,8,9,10,11,12]. In OPMD patients, the alanine residues increase from the normal range of 10–11 to 18 or more [13]. Consequently, understanding the role of RBPs in post-transcriptional mRNA processing is vital for elucidating the regulation of gene expression and uncovering the molecular mechanisms underlying human diseases [14].

Polyadenylation is a critical mRNA maturation process in nearly all eukaryotic organisms [15,16]. This process involves two primary steps: pre-mRNA cleavage and poly(A) tail formation. The poly(A) tail’s length is pivotal for the transport of mature mRNA to the cytoplasm, its translation efficiency at certain developmental stages, and mRNA quality control and degradation. Poly(A) RNA binding proteins (Pabs) are a class of proteins that can specifically bind to poly(A) RNA with high affinity and can regulate multiple steps of gene expression. Within this family, PABPN1 and PABPC1 are the most intensively studied Pabs and are located in the cytoplasm and nucleus, respectively [17,18].

PABPN1, a highly conserved nuclear protein in eukaryotes, and the *PABPN1* gene is located in the chromosome 14q11.2 [19]. PABPN1 has 306 amino acids and includes an alanine-rich N-segment, a proline-rich region, an RNA-binding domain, and a C-terminal domain. It is an abundant nuclear protein that has a high affinity for the poly(A) tail at the 3′ end of mRNA and is involved in the polyadenylation of mRNA [11]. PABPN1 is integral to RNA metabolism, regulating poly(A) tail length within the nucleus and thereby influencing key post-transcriptional processes, such as the addition of poly(A) tails to mRNA [20,21,22,23,24]. Alternative polyadenylation (APA) is a crucial regulatory mechanism for gene expression in eukaryotes. The same gene can produce transcripts with different lengths of 3′ UTR by selecting different poly(A) sites, thereby affecting the stability, subcellular localization, and translation efficiency of mRNA [25,26]. Studies have shown that PABPN1 is a regulator of the APA process [27,28,29]. Xiong et al. demonstrated that PABPN1 is an oncogenic APA factor in clear cell renal cell carcinoma (ccRCC) [30]. The RRM domain is the core region where PABPN1 binds to poly(A) RNA. This domain may confer on PABPN1 the ability to selectively recognize RNA molecules. Its RNA-binding ability affects the stability, transport, and degradation of mRNA, thereby regulating gene expression levels. The CC promotes polyadenylation by interacting with PAP, while the RRM domain ensures RNA binding. The CCRRM fragment of PABPN1 may ensure the accuracy and efficiency of mRNA 3′ tailing. Moreover, studies suggest that PABPN1 is essential for telomerase RNA biogenesis and telomere maintenance, as it promotes the maturation of telomerase RNA by appending long poly(A) tails [31]. However, the molecular mechanisms underlying the recognition and binding of poly(A) RNA by the RRM domain remain to be fully elucidated.

In this report, we employed SAXS to elucidate the solution structure of the CCRRM fragment in PABPN1 and used Electrophoretic Mobility Shift Assay (EMSA) and SHAPE to determine the sequence preferences of CCRRM-RNA binding. SAXS analysis indicated that CCRRM exists as a monomer in solution, while EMSA confirmed its effective interaction with A-rich sequences. Additionally, SHAPE revealed PABPN1′s affinity for sequences at the 3′ end of mRNA, showing high affinity for A-rich, moderate for GU-rich and CU-rich sequences, and negligible for AU-rich and CA-rich sequences. Comparative analysis of bead models of CCRRM and the CCRRM-A-rich complex demonstrated that the CCRRM fragment undergoes a conformational change upon RNA binding.

## 2. Materials and Methods

### 2.1. Sample Preparation

#### 2.1.1. Protein Purification

The CCRRM gene fargment was cloned into the pET-MG vector through the BamHI/XhoI (NEB, Ipswich, MA, USA) restriction sites. Recombinant plasmids were transferred into BL21 competent cells (Invitrogen, Carlsbad, CA, USA) under sterile conditions and cultured overnight at 37 °C and 225 rpm. A single colony was selected and inoculated into 4 mL of LB medium containing antibiotics and grown overnight at 37 °C. Cultures were then transferred to 1 L of pre-sterilized LB medium supplemented with antibiotics and grown to an OD600 of 0.6–0.8. Protein expression was induced with Isopropyl β-D-1-thiogalactopyranoside (IPTG) (Sangon Biotech, Shanghai, China) to a final concentration of 0.2 mM and incubated overnight at 22 °C and 225 rpm. Sample were harvested by centrifugation, and the pellet was lysed in lysis buffer (50 mM NaH_2_PO_4_, 150 mM NaCl, 10 mM Imidazole, pH 8.0) containing 1 mM PMSF. Cell lysates were clarified by centrifugation, and the supernatant was subjected to Ni-NTA affinity chromatography (Roche, Basel, Switzerland). Nonspecific proteins were removed using a wash buffer containing 50 mM imidazole (Sangon Biotech, Shanghai, China). The target protein was eluted with an elution buffer containing 250 mM imidazole. A final elution was performed using a strip buffer containing 500 mM imidazole to remove any remaining proteins. Protein expression and purification results were analyzed by SDS-PAGE [32]. Further purification was achieved using size exclusion chromatography Size-Exclusion Chromatography with Fast Protein Liquid Chromatography (SEC-FPLC).

#### 2.1.2. RNA Preparation

Previous studies have shown that PABPN1 binds to poly(A); therefore, we designed A-rich RNA containing poly(A) [28,33,34,35]. Cis-acting elements and trans-acting factors that regulate RNA degradation within the 3′ UTR include AU-rich elements [36,37], CA-rich elements [38], CU-rich elements, and GU-rich elements [39]. We designed RE_A-rich RNA containing poly(A) and multiple elements within the 3′ UTR. The sequences of A-rich and RE_A-rich are shown in Table 1. A-rich and RE_A-rich RNA plasmids were synthesized by Sangon Biotech (Shanghai, China). PCR amplification was used to generate templates for in vitro transcription. RNA was then produced using an in vitro transcription protocol, as described in previously published studies [40]. RNA was purified and separated using polyacrylamide gel electrophoresis with 8 M urea. 5′-biotin-modified A_6_, A_12_, and A_18_ RNAs were synthesized by Sangon Biotech (Shanghai, China).

### 2.2. SEC-FPLC

SEC-FPLC was performed using Superdex 75 10/300 GL (Cytiva, Marlborough, MA, USA). Protein samples were dissolved in tris buffer (25 mM Tris-HCl, 150 mM NaCl, pH 8.0) and applied to the columns to separate based on molecular size. Sample integrity was assessed by analyzing peak positions.

### 2.3. EMSA

The protein sample was incubated with RNA in a binding buffer (10 mM Tris-HCl, pH 8.0, 25 mM KCl, 10 mM NaCl, 1 mM MgCl_2_, 10% glycerol, 0.5 mM DTT) at 25 °C for 20 min [33]. Complex formation was confirmed by 12% Native-PAGE gel electrophoresis [41,42]. The results are visualized by staining the gel with the nucleic acid dye 4SGelred, 10,000× in water (Sangon Biotech, Shanghai, China).

### 2.4. Biolayer Interferometry [43]

Biolayer interferometry (BLI), a label-free technique, was employed to measure biomolecular interactions. A_6_, A_12_, and A_18_ were immobilized on High Precision Streptavidin biosensors (Sartorius, Göttingen, Germany). Subsequently, the RNA conjugated to the biosensor tips was allowed to interact with varying concentrations of CCRRM protein in solution. The Octet^®^ RH96 system was utilized to monitor these interactions in real time, thereby elucidating the affinity, association, and dissociation rates of CCRRM protein with A_6_, A_12_, and A_18_ RNA.

### 2.5. SAXS Data Collection and Analysis

SAXS data were collected at the BL 19U2 beamline of the Shanghai Synchrotron Radiation Facility (SSRF). Samples were prepared in Tris-HCl buffer and analyzed using SEC-SAXS. Data were collected at a flow rate of 0.5 mL/min, with images captured at a rate of 40 frames per minute. Data processing was performed using RAW software (Version 2.1.4), and the folding status of the samples was assessed using a Kratky plot [44,45,46,47].

### 2.6. SHAPE Probing Analysis

Protein and RNA samples were prepared and incubated according to the EMSA protocol. Unreacted proteins were removed by phenol-chloroform extraction (Phenol: chloroform: isoamyl alcohol = 25: 24: 1). Next, 9 µL of RNA samples (divided into sequencing and control groups) and 9 µL of RNA-protein complex were aliquoted into separate tubes. 1 µL of DMSO and 1 µL of 1M7 (1-methyl-7-nitroisatoic anhydride) were added to tubes and incubate at 35 °C for 10 min. After incubation, add 500 µL of ethanol precipitation buffer (75% ethanol, 400 µL 5 M NaCl, 40 µL 0.5 M EDTA, 9 mL ultrapure water) and 1.5 µL of GlycoBlue (Invitrogen, Carlsbad, CA, USA) to each tube. Subsequently, all samples were placed in a −80 °C refrigerator for about 1 h to allow RNA to fully precipitate, and then centrifuged at 14,500 rpm for 45 min. After centrifugation, the supernatant was discarded, and the tube was inverted to completely dry the precipitate (drying is indicated by the fading of the blue color of the precipitate). Next, 9 µL of ultrapure water was added to gently resuspend the precipitate, followed by the addition of 0.5 µL 100 µM FAM primer (FAM-GAACCGGACCGAAGCCCG) to all tubes. The reaction was performed at 65 °C for 5 min, then at 35 °C for 5 min. After cooling to 4 °C, RT mix (a mixture of reverse transcriptase buffer and dNTPs in a 5:1 ratio) was added. For the sequencing group, ddA was added, and the mixture was thoroughly mixed and kept at 4 °C for 5 min. The sample was then heated to 49 °C, 1 µL of superScript™ III reverse transcriptase (Invitrogen, Carlsbad, CA, USA) was added, and the reaction was incubated for 30 min. The temperature was then increased to 95 °C, 1 µL of 4 M NaOH was added to remove excess RNA, and the reaction was continued for 5 min. Finally, 29 µL of acid stop dye was added, and the reaction was continued at 95 °C for 5 min to terminate the reaction. The samples were sent to Sangon Biotech for sequencing and the results were analyzed using ShapeFinder (Version 1.0) [48,49,50].

## 3. Results

### 3.1. Domain Structure of PABPN1 and Preparation of CCRRM Protein Sample

**Figure 1:**
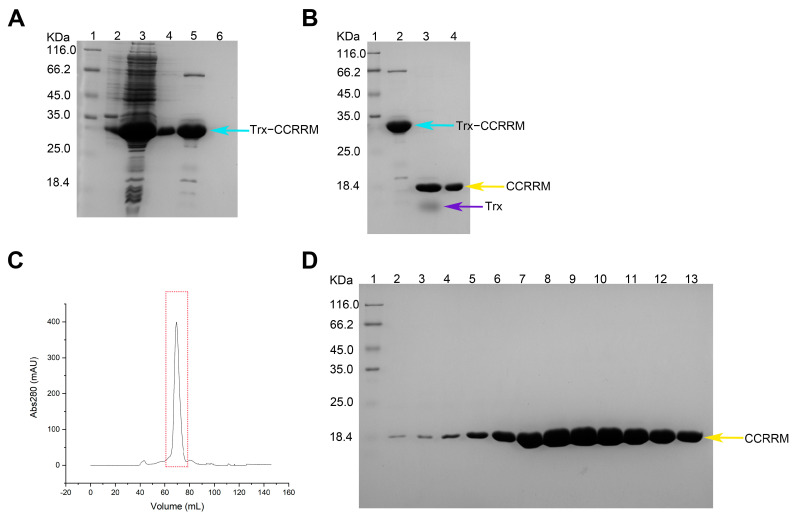
Expression and purification of CCRRM fragment of PABPN1. The cyan, yellow, and purple arrows represent the samples of Trx-CCRRM, CCRRM and Trx, respectively. (**A**) Ni-NTA purification of CCRRM. Lane 1, Unstained protein marker; lane 2, precipitation after ultrasonic cell lysis; lane 3, supernatant after ultrasonic cell lysis; lane 4, sample washed with wash buffer; lane 5, sample washed with elution buffer; lane 6, sample washed with strip buffer. (**B**) Enzyme digestion. Lane 1, Unstained protein marker; lane 2, sample before enzyme digestion; lane 3, sample after enzyme digestion; lane 4, sample that passed through the nickel column after enzyme digestion. (**C**) SEC-FPLC profile of CCRRM. The peak corresponding to the target protein is outlined in a red box. (**D**) SDS-PAGE analysis of protein samples isolated from the peak positions as illustrated in Figure C. Lane 1, Unstained protein marker; lanes 2–13, the samples of the peak of SEC-FPLC.

First, we aim to analyze structure and conformations of the PABPN1 to understand its structure and function. We designed and constructed a recombinant plasmid that contains the CC and RRM domain (Figure S1A). And we utilized https://alphafoldserver.com/ (accessed on 28 August 2024) to predict the three-dimensional structure of CCRRM fragment and validated the model’s rationality. We selected the known RRM structures with PDB IDs 3B4D and 3UCG and aligned them with the CCRRM structure predicted by AlphaFold3 (Figure S1C). We found that the root-mean-square deviations (RMSDs) were 0.427 and 0.428, respectively, indicating that the result predicted by AlphaFold3 has high accuracy. CCRRM fragment contains 141 amino acids and is primarily composed of four α-helices and five β-sheets and nine loops (Figure S1B). The theoretical molecular weight of the CCRRM and the Trx tag were calculated to be 15.8 kDa and 12 kDa, respectively. The result of the Ni-NTA affinity purification is shown in Figure 1A. The target fusion protein band, indicated by the cyan arrow, migrates between 25.0 kDa and 35.0 kDa, corresponding to the expected molecular mass of 27.8 kDa.

Following affinity purification, thrombin cleavage was performed to remove the Trx tag. The sample was incubated with thrombin overnight, and then re-applied to nickel affinity column for further purification. The eluted fractions were analyzed by SDS-PAGE (Figure 1B). The yellow arrows in the gel indicate CCRRM, which migrates at approximately 18 kDa, consistent with its theoretical molecular weight of 15.8 kDa.

Finally, SEC-FPLC was used to further purify the CCRRM sample. The SEC-FPLC profile, shown in Figure 1C, reveals a distinct peak, and the corresponding fractions were collected and analyzed by SDS-PAGE (Figure 1D). The gel analysis shows a clear, single band at approximately 18.4 kDa, consistent with the theoretical molecular weight of the CCRRM fragment.

In summary, the recombinant CCRRM protein was purified to >90% purity through a three-step process, including Ni-NTA affinity chromatography, thrombin cleavage, and SEC-FPLC.

### 3.2. The CCRRM’s Conformation in Solution Remains Stable Irrespective of Concentration and Interact with A-Rich RNA

**Figure 2:**
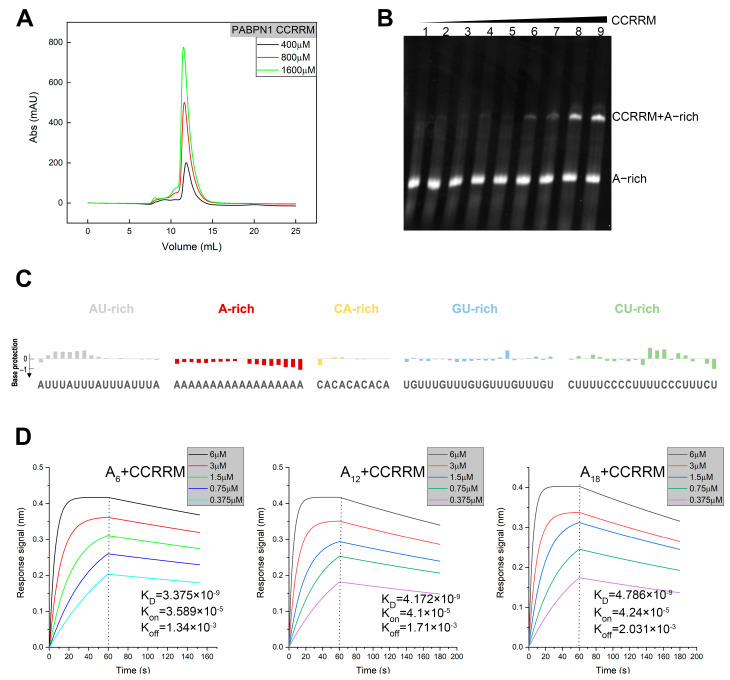
Analysis of the homogeneity and conformation of the CCRRM and binding affinity of CCRRM for A-Rich RNA. (**A**) SEC-FPLC analysis of the CCRRM at different concentrations. Black, red and green represent the states at concentrations of 400 µM, 800 µM and 1600 µM, respectively. (**B**) Analysis of the interaction CCRRM with A-rich RNA. The concentration of A-rich RNA is 50 µM. Lanes 1–9 represent the protein-RNA molar concentration ratio of 0, 0.1, 0.15, 0.2, 0.25, 0.5, 1, 3, 5, respectively. (**C**) SHAPE analysis of RE_A-rich in the presence of CCRRM. Residues are indicated on the X-axis. The height of the bar graph represents the degree of base protection after binding to the CCRRM protein. The AU-rich, A-rich, CA-rich, GU-rich, CU-rich and A-rich of RNA are labeled in gray, red, orange, blue and green, respectively. (**D**) BLI analysis of the binding affinity between CCRRM and A-rich RNA. BLI sensorgrams were generated using 0.5 µM 5′-biotin RNA conjugated to streptavidin-loaded biosensors, followed by a range of CCRRM concentrations to ascertain the affinity the binding (K_on_) and dissociation (K_off_) rate constants between RNA and CCRRM. Interaction analysis results of CCRRM with A_6_, A_12_, and A_18_ from left to right.

To investigate the structural and functional properties of CCRRM in solution, we systematically prepared CCRRM protein samples at various concentrations. The homogeneity of CCRRM was evaluated using size exclusion chromatography with a Superdex Increase 75 10/300 GL column (Figure 2A). Our result showed that the elution peak was consistent across different protein concentrations, indicating a high degree of uniformity for CCRRM in solution. Next, to explore the interaction between CCRRM and RNA, we designed and synthesized A-rich and U-rich RNA (Table 1). EMSA was utilized to detect the interactions between CCRRM and U-rich and A-rich RNAs. CCRRM did not bind to U-rich RNA, but did bind to A-rich RNA (Figure S2 and Figure 2B). Additionally, as the concentration of CCRRM increased, the RNA-protein complex band (upper band) was significantly enhanced.

Having demonstrated that the CCRRM fragment of PABPN1 binds to A-rich sequence, we next sought to explore its interactions with other mRNA 3′ UTR elements. To probe these interactions, we designed RNA construct containing A-rich, AU-rich, CU-rich, GU-rich, and CA-rich sequences for SHAPE analysis, with construct flanked by a standard SHAPE sequence (Table 1). We employed SHAPE analysis to map the binding sites of CCRRM on the RE_A-rich RNA. We evaluated the effects of CCRRM on RNA conformation by comparing SHAPE reactivity in the absence and presence of the protein (Figure S3 and Figure 2C). The downward bars in Figure 2C signify base protection due to protein binding. SHAPE reactivity values of GU-rich (blue bars) remained stable in both protein-free and protein-bound states. In contrast, the A-rich (red bars) exhibited higher SHAPE reactivities in the protein-free state, indicating higher conformational dynamics in the absence of protein. Upon CCRRM binding, the SHAPE reactivity of CA-rich (orange bars) and GU-rich (blue bars) remained stable, while the A-rich sequence showed obvious protection. Additionally, SHAPE reactivity of AU-rich sequences increased, whereas that of CU-rich sequences decreased, suggesting partial protection of CU-rich sequences and increased flexibility of AU-rich sequences upon CCRRM binding.

We further investigated the interactions between CCRRM and A-rich RNAs. We synthesized 5′-biotin-modified A_6_, A_12_, and A_18_ RNAs and employed BLI to analyze their binding to CCRRM. High Precision Streptavidin biosensors were incubated with these RNAs to immobilize them on the sensor surface. The RNA-immobilized sensors were then incubated with CCRRM protein at room temperature across a range of protein concentrations. Utilizing the Octet^®^BLI system, we monitored the binding events in real time, determining the association rate (K_on_), dissociation rate (K_off_), and equilibrium dissociation constant (K_D_) (Figure 2D). The K_D_ values of A_6_, A_12_, and A_18_ RNAs were 3.375 × 10^−9^ M, 4.172 × 10^−9^ M, and 4.786 × 10^−9^ M, respectively, indicating the highest affinity for A_6_ RNA and the lowest for A_12_ RNA, with A_18_ RNA intermediate. The K_on_ values were 3.589 × 10^−5^ Ms^−1^, 4.1 × 10^−5^ Ms^−1^, and 4.24 × 10^−5^ Ms^−1^, respectively, showing the fastest binding with A_18_ RNA, slightly slower with A_12_ RNA, and slowest with A_6_ RNA. The K_off_ values were 1.34 × 10^−3^ s^−1^, 1.71 × 10^−3^ s^−1^, and 2.031 × 10^−3^ s^−1^, respectively, confirming the fastest dissociation for A_18_ RNA, followed by A_12_ RNA, and slowest for A_6_ RNA.

### 3.3. A-Rich RNA Induces Conformational Changes in CCRRM, Primarily Occurring in the CC Region

**Figure 3:**
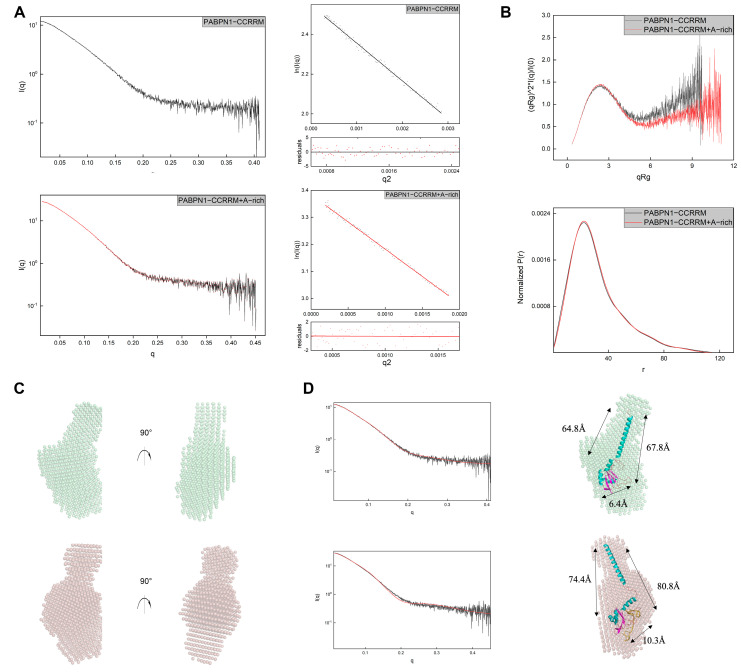
A-rich RNA binds to the CCRRM and induces conformational changes. (**A**) Experimental scattering profiles and Guinier plots are shown for the CCRRM in the absence and presence of A-rich RNA. The scattering profiles and Guinier plot are displayed on the left and right, with the RNA-free CCRRM shown in the upper series and the RNA-bound CCRRM in the lower series. (**B**) Kratky plot (upper) and normalized P(r) analysis (lower) of the CCRRM. The RNA-free CCRRM is represented in black, while the RNA-bound CCRRM is depicted in red. (**C**) Low-resolution bead models calculated by DAMMIF from the SAXS data. The RNA-free CCRRM (upper series) is shown in pale green, and the RNA-bound CCRRM is represented in dark salmon. (**D**) Predicted 3D models were docked into the SAXS bead models. The theoretical scattering curve of the CCRRM predicted structure (red) was compared to the experimental scattering curves (black) by CRYSOL. The coloring of the bead models of the RNA-free CCRRM (upper series) and RNA-bound CCRRM (lower series) is consistent with Figure 3C. The theoretical scattering curve (red) derived from the predicted 3D model is mapped with experimental SAXS data plotting by CRYSOL, with RNA-free: χ^2^ = 1.55 and RNA-bound: χ^2^ = 1.98. In the RNA-free CCRRM state, the dimensions for the top loop1 and bottom loop9, β1 and β5, and α1 and β1 are 64.8, 6.4, and 67.8, respectively. In the RNA-bound CCRRM state, the dimensions for the top loop1 and bottom loop9, β1 and β5, and α1 and β1 are 74.4, 10.3, and 80.8, respectively.

Having established the sequence-specific binding affinity of CCRRM for A-rich RNA, we proceeded to investigate the conformational changes in CCRRM in the absence or presence of RNA using SAXS. The SAXS profiles of CCRRM with and without RNA are shown in Figure 3. Based on SAXS analysis, the Guinier radius of gyration (Rg) without RNA is about 23.75, which is slightly smaller than the Rg of 24.61 in the presence of RNA. Paired-distance (P(r)) distributions were calculated from the SAXS profiles (Figure 3A,B; Table 2). The Kratky plots of CCRRM with and without RNA showed that RNA binding improved the folding of CCRRM, suggesting a structural stabilization effect.

The 3D structures were predicted by AlphaFold3 and then fitted to the SAXS experimental data to reconstruct the CCRRM structures in both RNA-free and RNA-bound states (Figure 3C). The resulting 3D-predicted model matched the angles and dimensions of the bead model calculated with SAXS. The CRYSOL results showed that the RNA-free and RNA-bound models of the CCRRM agreed well with the experimental scattering profile (chi^2^ = 1.55 and 1.98, respectively) (Figure 3D left). Comparative analysis revealed that A-rich RNA binding induced conformation changes in CCRRM (Figure 3D right). In the RNA-free CCRRM state, the dimensions for the top Loop1 and bottom Loop9, β1 and β5, and α1 and β1 are 64.8, 6.4, and 67.8, respectively (Figure 3D upper model). In the RNA-bound CCRRM state, the dimensions increased to 74.4, 10.3, and 80.8 (Figure 3D lower model).

**Figure 4:**
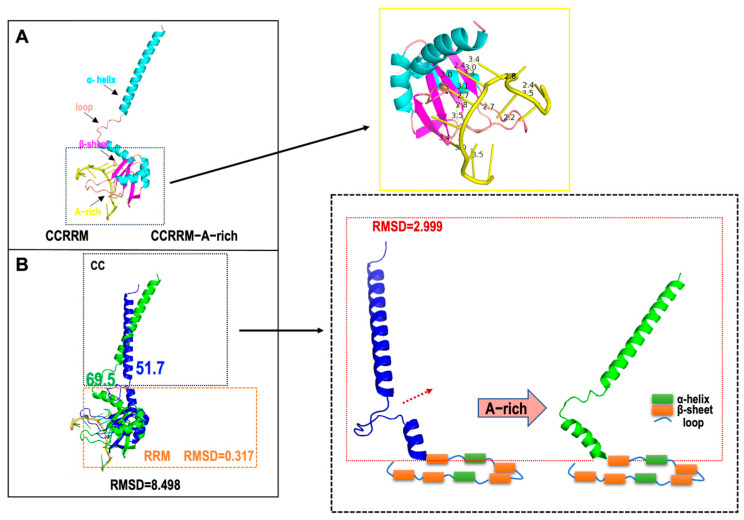
Local rearrangements diagram of A-rich binding. (**A**) The 3D structure of CCRRM and CCRRM-A-rich. A-rich is marked in yellow. The yellow solid line box highlights the interaction between CCRRM and A-rich RNA. (**B**) The overall alignment of the 3D structures of CCRRM and CCRRM-A-rich shows that the RMSD is 8.498. The dotted box on the right shows a magnified view of the CC region. The red dotted box highlights the conformational changes in the CC before and after RNA binding. The RRM region is simplified using rectangles and lines, where the orange rectangle represents the beta sheet, the green rectangle represents the alpha helix, and the line represents the loop.

Subsequently, we aligned the three-dimensional structures of CCRRM before and after RNA binding (Figure 4). The yellow solid line box in Figure 4A highlights the distances between CCRRM residues and RNA bases. Which range from 2 to 4 Å, indicating that the interactions are predominantly hydrogen bonds and van der Waals forces. RNA primarily binds to the RRM domain, while the major conformational changes occur in the CC region. The angle between the CC and the RRM domain increased from 51.7° to 69.5° (Figure 4B). The RMSD of the overall alignment between the two states was 8.498, and the RMSD for the RRM domain was 0.317, further indicating that RNA binding mainly affects the CC. The RMSD of the CC region (highlighted in the dotted box in Figure 4B) was 2.999, confirming that RNA binding induces conformational changes in CCRRM, primary in the rotating CC.

## 4. Discussion

PABPN1 is a crucial RNA-binding protein involved in post-transcriptional RNA processing. Previous research has demonstrated that PABPN1 is multifunctional, playing roles in mRNA 3′ end processing, nonsense-mediated mRNA decay, and mRNA transport [28,51,52,53,54,55]. It stimulates the poly(A) polymerase (PAP) activity by binding to the nascent poly(A) tail and participates in the polyadenylation process along with cleavage and polyadenylation specificity factor (CPSF) [28]. Furthermore, PABPN1 is involved in the polyadenylation of transcripts containing retained introns and RNA exosome-mediated degradation [3]. PABPN1 also regulates gene expression by affecting alternative polyadenylation, particularly at the interdependence between 3′ intron splicing, 3′ end processing, and polyadenylation [56]. Mutations in the PABPN1 gene can lead to OPMD, a specific form of muscular atrophy. Therefore, studying the structure and function of PABPN1′s CCRRM fragment is of significant importance.

In this study, we analyzed the solution state of CCRRM using SEC-FPLC. The results demonstrated that the conformation of CCRRM is independent of protein concentration, maintaining high stability at both low and high concentrations, indicating its high homogeneity in solution. SAXS analysis revealed that the CCRRM fragment of PABPN1 exists as a monomer, contradicting previous studies suggesting that the RRM domain exists as a dimer [57]. These results imply that PABPN1 may adopt different assembly states depending on the domain, which is crucial for understanding its functional diversity. The CCRRM fragment may play a unique role in PABPN1′s RNA binding and regulatory functions.

PABPN1 is well known for its specific binding to poly(A) RNA, and our study expands on this function [58,59]. PABPN1 also modulates alternative polyadenylation [60,61], demonstrating that PABPN1 regulates 3′ UTR length which could influence downstream post-transcriptional regulatory mechanisms. Analysis of the solution-binding conformation of CCRRM with A-rich RNA revealed that RNA primarily interacts with the α-helix and β-sheet regions of CCRRM, which are structurally similar to the RRM domain [57,62,63]. This suggests that the primary RNA-binding domain of PABPN1 is the RRM [64], while the CC may serve an auxiliary role. However, the precise function and molecular mechanism of the CC remain to be elucidated. SHAPE experimental results showed that the CCRRM fragment can bind not only to A-rich RNA but also to AU-rich and CU-rich elements in the 3′ UTR of mRNA, potentially affecting mRNA stability and translation efficiency [65]. While SHAPE is a useful tool for identifying RNA-flexibility and inferring potential protein–RNA interactions, it does not provide high-resolution details about the nature or specificity of these interactions [66,67]. In this study, we use SHAPE to demonstrate the interaction between CCRRM and RNA, but the method does not allow us to determine the specific binding sites. Further investigation using structural methods such as CLIP-seq, NMR, or crystallography may be required to resolve the exact interaction interface.

Our experiments demonstrated that the binding affinity of CCRRM for A-rich RNA is influenced by the number of adenines, with A_6_ exhibiting the highest affinity, followed by A_12_ and A_18_. The binding rate increases with the number of adenines (A_6_ < A_12_ < A_18_), while the dissociation rate shows the opposite pattern (A_18_ > A_12_ > A_6_) This reveals a subtle regulatory mechanism underlying PABPN1′s RNA-binding specificity, which may have significant implications for mRNA processing and degradation [68]. This variation in binding affinity may be closely associated with the secondary structure and dynamics, a hypothesis that requires further validation through structural biology studies. Additionally, BLI measurements have an inherent error range, with intra-experimental repeatability errors typically ranging from 10% to 20%. Therefore, a difference of less than 20% may fall within the normal experimental error range and may not conclusively indicate a significant difference in affinity. This limitation suggests the need for cross-validation with other experimental methods in the future.

The distances between the amino acid residues of CCRRM and the bases of the A-rich fall within the range of hydrogen bond distances (2–3 Å) and van der Waals forces (3–4 Å) [63], indicating that the interactions between CCRRM and RNA are predominantly hydrogen bonds and van der Waals forces. The binding modes of the known PDB IDs 4EGL and 1X4G RRM-RNA complexes also involve hydrogen bonding. CCRRM may bind RNA through a similar interaction mode. Upon RNA binding, the Rg value is slightly higher than in the unbound state, suggesting that the CCRRM protein may undergo local domain rearrangement. A further analysis of conformational changes before and after RNA binding suggests that the primary structural adjustment of the CCRRM protein is concentrated in the CC region and the CC undergoing rotation upon A-rich RNA binding. After RNA binding, the angle between the CC and RRM domains increases, indicating that the binding site may experience conformational opening. This local conformational adjustment may increase the stability of RNA binding, thereby enhancing binding specificity.

Finally, we observed significant conformational changes in CCRRM upon binding to A-rich RNA. This observation aligns with the dynamic properties of PABPN1 during RNA binding and may relate to the regulatory mechanisms of RNA processing and degradation. These conformational changes might also influence PABPN1′s interactions with other RNA-binding proteins, thereby modulating the overall fate of mRNA.

## 5. Conclusions

In this study, we investigated the solution structure of the CCRRM fragment (residues 114–254) and its interaction with RNA. First, we designed and purified the recombinant CCRRM protein. Next, EMSA and BLI confirmed that CCRRM exhibits high affinity for poly(A) RNA. SHAPE chemical probing demonstrated that CCRRM binds poly(A) RNA with high affinity, exhibits moderate affinity for GU-rich and CU-rich sequences, and shows negligible binding to AU-rich and CA-rich sequences. Finally, by combining 3D modeling and SAXS analysis, we analyzed the solution conformations of CCRRM in its free and RNA-bound states. The results revealed that RNA binding induces conformational changes in CCRRM, primarily within the CC region.

## Figures and Tables

**Table 1:** Sequences of A-rich and RE_A-rich RNA.

RNA Name	Sequence
A-rich	UAAUACGACUCACUAUAGGGUGGUCAGUCGAGUGGAAAAAA AAAAAAAAAAAAGGGCGGCAUGGUCCCAGCCUCCU
RE_A-rich	CCUUCGGGCCAAAUUUAUUUAUUUAUUUAUUUAGCUGACGA UAAAAAAAAAAAAAAAAAACCACACACACAGGAAUCGACUC UGUUUGUUUGUGUUUGUUUGUACUGAAUUGGCACUUUUCCC CUUUUCCCUUUCUGGACUGGCAUCGAUCCGGUUCGCCGGAUC CAAAUCGGGCUUCGGUCCGGUUC

**Table 2:** Radius of gyration (Rg) and maximum dimension (Dmax) of the CCRRM in RNA-free and RNA-bound solution conditions were determined using SAXS.

**Structural Parameters**	**RNA-Free**	**RNA-Bound**
I (0) (cm^−1^) from Guinier fit	12.69 ± 0.03	29.48 ± 0.06
Rg (Å) from Guinier fit	23.75 ± 0.1	24.61 ± 0.11
Rg (Å) from P(r)	26.31 ± 0.34	26.1 ± 0.18
Dmax (Å) from P(r)	119.0	114.0
I (0) (cm^−1^) from P(r)	13.04 ± 0.06	29.82 ± 0.08

## Data Availability

The original contributions presented in this work are included in this article; further inquiries can be directed to the corresponding author.

## Supplementary Materials

The following supporting information can be downloaded at: https://www.mdpi.com/article/10.3390/biology14040432/s1. File S1: full western blots in Figure 1 and Figure 2; File S2: Figure S1. Domain structure of CCRRM fragment of PABPN1. Figure S2. Analysis of the interaction CCRRM with U-rich RNA. Figure S3. SHAPE analysis of RE_A-rich.

## Abbreviations

PABPN1	Poly(A) Binding Protein Nuclear 1
CC	coiled-coil domain
RRM	RNA recognition motif
SAXS	small-angle X-ray scattering
SHAPE	selective 2′-hydroxyl acylation analyzed by primer extension
RBPs	RNA-binding proteins
OPMD	oculopharyngeal muscular dystrophy
Pabs	Poly(A) RNA binding proteins
APA	Alternative polyadenylation
ccRCC	clear cell renal cell carcinoma
EMSA	Electrophoretic Mobility Shift Assay
IPTG	Isopropyl β-D-1-thiogalactopyranoside
SEC-FPLC	Size-Exclusion Chromatography with Fast Protein Liquid Chromatography
BLI	Biolayer interferometry
SSRF	Shanghai Synchrotron Radiation Facility
RMSDs	root-mean-square deviations
1M7	1-methyl-7-nitroisatoic anhydride
Rg	Radius of gyration
Dmax	maximum dimension
CPSF	cleavage and polyadenylation specificity factor
