# Supplementary material for: RNA Binding to CCRRM of PABPN1 Induces Conformation Change
Source: Biology (Basel). 2025 Apr 17;14(4):432. doi: 10.3390/biology14040432 (PMC12024694; doi:10.3390/biology14040432)
Supplement: Supplementary file 1 [file biology-14-00432-s001.zip › biology-3561413-figures.pdf]

Supplementary information

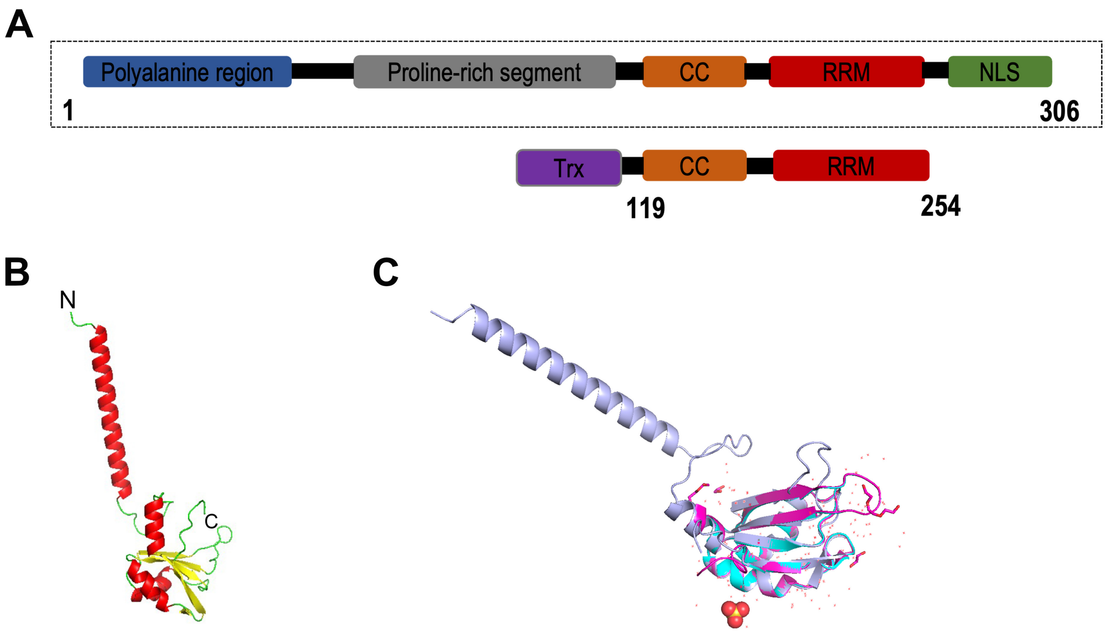

Figure S1 Domain structure of CCRRM fragment of PABPN1. (A) The structure of PABPN1 is shown in the upper box. It comprises the N-terminal polyalanine region, a proline-rich segment, a coiled-coil domain, an RNA-binding domain, and a C-terminal nuclear localization signal and displays them in dark blue, gray, orange, red and green respectively. The bottom is the target domain CCRRM. The purple shows the Trx, the orange and red show the CC and RRM domain respectively. (B) 3D structure of CCRRM predicted by AlphaFold3. The secondary structure is shown in different color. The α-helix, β-sheet and loop are labeled in red, yellow and green, respectively. (C) Alignment of the known RRM structures of PDB ID 3B4D and 3UCG with the CCRRM structure predicted by Alphafold3. The structures 3B4D, 3UCG, and CCRRM are represented by blue, magenta, and light purple, respectively, with RMSD values of 0.427 and 0.428 for the alignments.

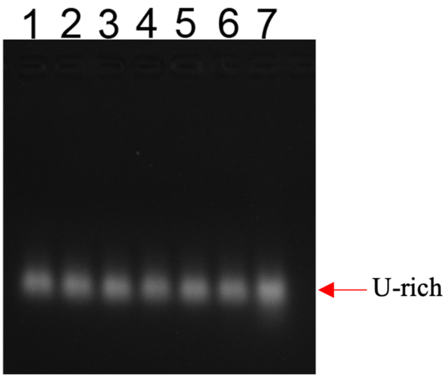

Figure S2 Analysis of the interaction CCRRM with U-rich RNA (Sequence: 5′-UAAUACGACUCACUAUAGGGUGGUCAGUCGAGUGGUUUUUUUUUUUUUUUUUUGGGCGGCAUGGUCCCAGCCUCCU-3′). The concentration of U-rich RNA is 40 μM. Lanes 1-7 represent the protein-RNA molar concentration ratio of 0, 0.5, 1, 2, 4, 6, 10, respectively.

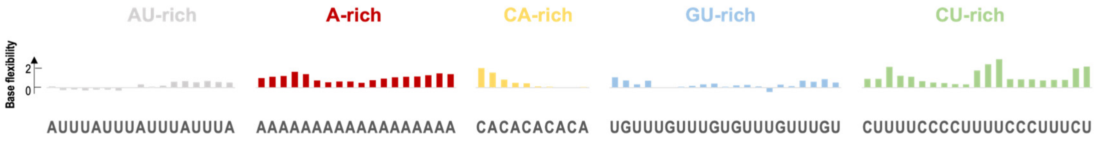

Figure S3 SHAPE analysis of RE_A-rich. Residues are indicated on the X-axis. The colored bars represent base flexibility. The AU-rich, A-rich, CA-rich, GU-rich, CU-rich and A-rich of RNA are labeled in gray, red, orange, blue and green, respectively. Upward bars represent base flexibility.
