# Supplementary figures and images for: RNA Binding to CCRRM of PABPN1 Induces Conformation Change
Source: Biology (Basel). 2025 Apr 17;14(4):432. doi: 10.3390/biology14040432 (PMC12024694; doi:10.3390/biology14040432)

Figure 1C and 1D

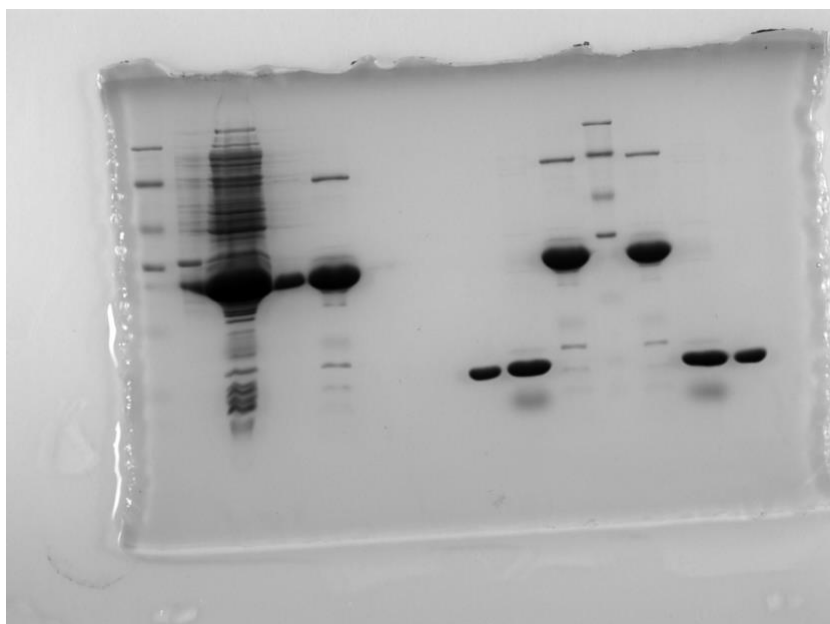

Figure 1F

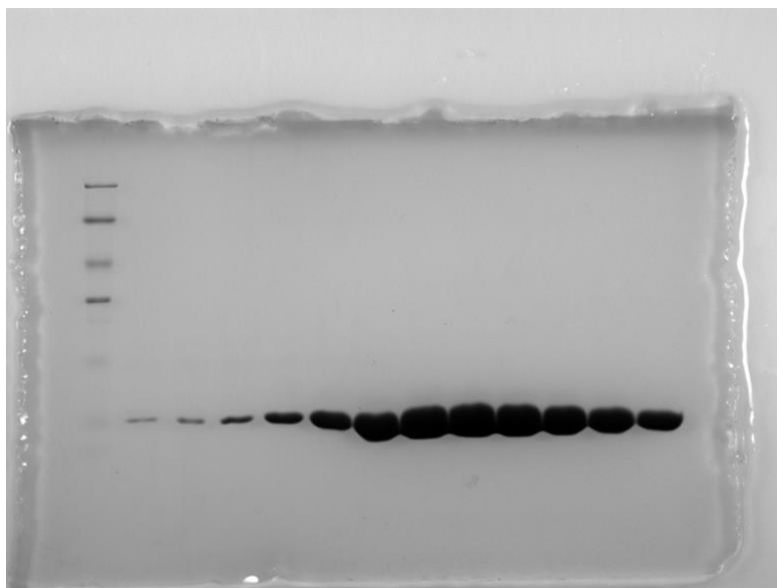

Figure 2B

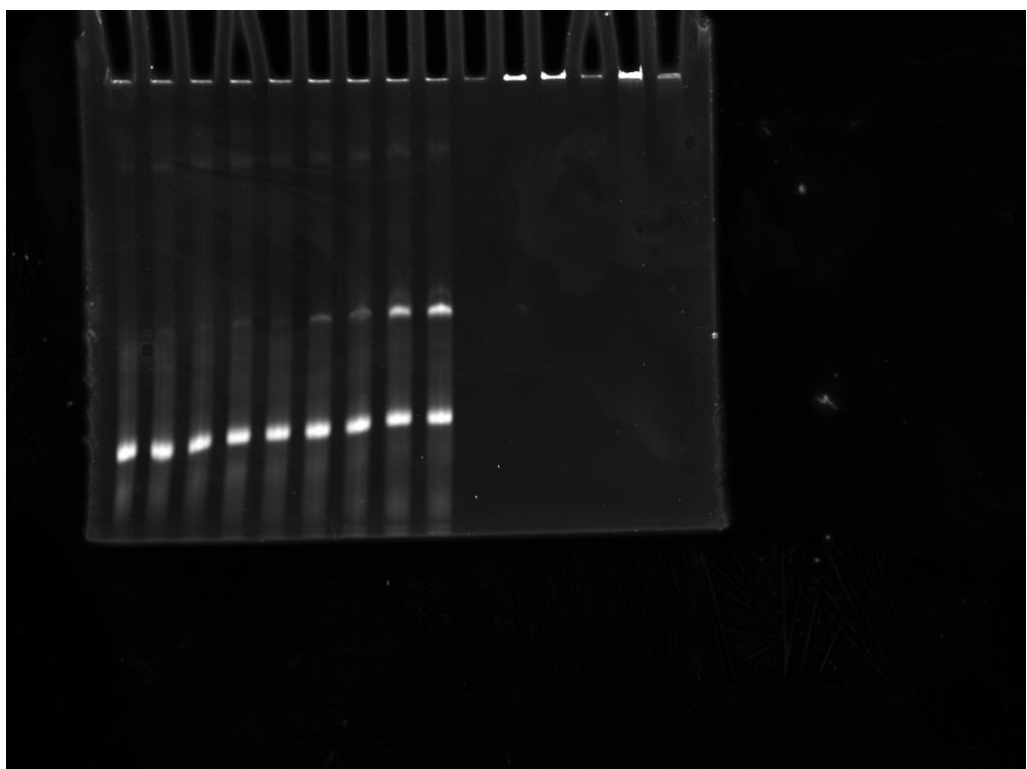

Supplement: Supplementary file 1 [file biology-14-00432-s001.zip › biology-3561413-western blots.pdf]
